# Jaagsiekte Sheep Retrovirus Biology and Oncogenesis

**DOI:** 10.3390/v2122618

**Published:** 2010-12-03

**Authors:** Andrew Hofacre, Hung Fan

**Affiliations:** Department of Molecular Biology and Biochemistry, Cancer Research Institute, University of California, Irvine, CA 92697, USA; E-Mail: ahofacre@uci.edu

**Keywords:** Jaagsiekte sheep retrovirus, JSRV, transformation, Env, Rej, ovine pulmonary adenocarcinoma, cancer

## Abstract

Jaagsiekte sheep retrovirus (JSRV) is the causative agent of a lung cancer in sheep known as ovine pulmonary adenocarcinoma (OPA). The disease has been identified around the world in several breeds of sheep and goats, and JSRV infection typically has a serious impact on affected flocks. In addition, studies on OPA are an excellent model for human lung carcinogenesis. A unique feature of JSRV is that its envelope (Env) protein functions as an oncogene. The JSRV Env-induced transformation or oncogenesis has been studied in a variety of cell systems and in animal models. Moreover, JSRV studies have provided insights into retroviral genomic RNA export/expression mechanisms. JSRV encodes a *trans*-acting factor (Rej) within the *env* gene necessary for the synthesis of Gag protein from unspliced viral RNA. This review summarizes research pertaining to JSRV-induced pathogenesis, Env transformation, and other aspects of JSRV biology.

## 1. Introduction

Ovine pulmonary adenocarcinoma (OPA) is a contagious neoplasm affecting sheep lungs [1–3]. The disease has been described in various breeds of sheep and also rarely in goats, but it does not affect cattle or other animals [4]. OPA is found in sheep in Europe, Africa, the Americas, and Asia. In the U.K. and South Africa, OPA accounts for almost 70% of all sheep tumors [4]. The disease is characterized by fluid build up in the lungs, resulting from the production of excess secretions by the tumor cells. OPA tumors are derived from secretory epithelial cells of the lungs, type II pneumocytes or Clara cells [5–9]. A field test for OPA, known as the wheelbarrow assay, is performed by elevating the body cavity of the sheep over the mouth, at which point lung fluid drains from the nose of infected sheep (Figure 4A). While this assay has been important for diagnosing OPA, the lung fluid from infected sheep also has been invaluable experimentally. Filtered lung fluid from infected sheep is able to reproduce the disease when inoculated intratracheally into unaffected sheep, indicating that there is a viral etiology to this disease [2,10,11]. While practical efforts have focused on controlling the disease, our current understanding of OPA has benefited from dissecting the molecular and biochemical aspects of the causative agent, jaagsiekte sheep retrovirus (JSRV). This review examines JSRV/OPA in the context of retroviral pathogenesis.

## 2. Retroviruses: Features, Genomic Organization, and Life Cycle

**Figure 1:**
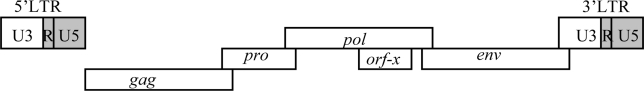
Genomic organization of JSRV. The JSRV genome is that of a betaretrovirus, possessing only the minimal number of genes required to carryout the retrovirus lifecycle; *gag*, *pro*, *pol*, and *env*. Uniquely, JSRV also has an additional open reading frame, *orf-x*, of unknown function. The boxes represent ORFs. LTR; long terminal repeats [2].

**Figure 2:**
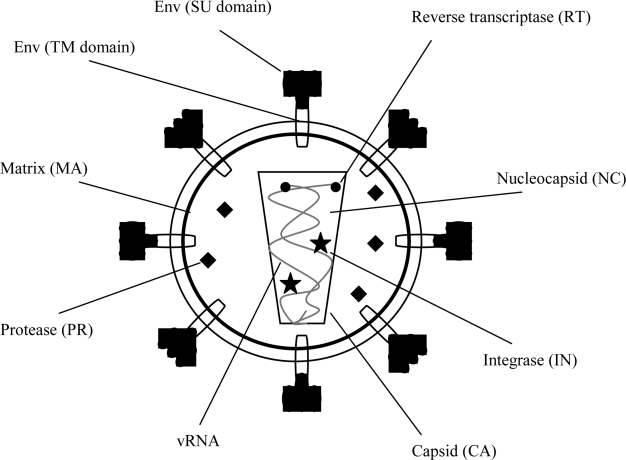
The structure of a typical retrovirus. All retroviruses carry two copies of their RNA genome (vRNA) and are composed of the viral structural proteins (SU, TM, MA, NC, and CA) and the viral enzymes (RT, PR, and IN).

Jaagsiekte sheep retrovirus (JSRV) is the causative agent of OPA. Retroviruses are a diverse family of RNA viruses that share common traits including structure, composition, and replication strategy [12]. Retroviruses have a spherical to conical capsid (depending on the virus) surrounded by an envelope, typically studded with spikes composed of virus-encoded glycoproteins [13]. This core (capsid) contains the positive-stranded viral RNA genome (7–12 Kb) along with virus-encoded enzymes (Figure 1). Retroviruses are broadly divided into two categories, simple and complex, based on the organization of their genomes [14]. JSRV, classified as a betaretrovirus, resembles a simple retrovirus, as its genomic organization contains only the essential genes characteristic of retroviruses: *gag*, *pro*, *pol*, and *env* (Figure 2). The *gag* gene encodes several structural proteins necessary to encapsidate the viral RNA genome and form the viral particle core, including nucleocapsid (NC), matrix (MA), and capsid (CA). The *pol* gene is located adjacent to or overlaps with *gag* and encodes enzymes that are essential during the viral life cycle, including reverse transcriptase (RT) and integrase (IN). The viral *pro* gene encodes viral protease that acts late in viral assembly and budding, allowing maturation of the viral particle [12]. The *env* gene encodes the surface (SU) and transmembrane (TM) domains of the envelope protein (Env) that is embedded in the membrane; Env protein binds the cell receptor during infection. For JSRV an additional open reading frame, *orf-x*, overlaps *pol* and has some unusual features, including a codon usage different from that of other genes within JSRV, and a very hydrophobic predicted amino-acid sequence that shows no similarities with any other known protein, with the exception of a low homology to a G protein-coupled receptor [15,16]. The role of this open reading frame is unknown. Finally, the RNA genome also contains non-encoding sequences that are unique to the 5’ end (the U5 region) and the 3’ end (the U3 region) which are in turn flanked by short repeated sequences (R) [12].

**Figure 3:**
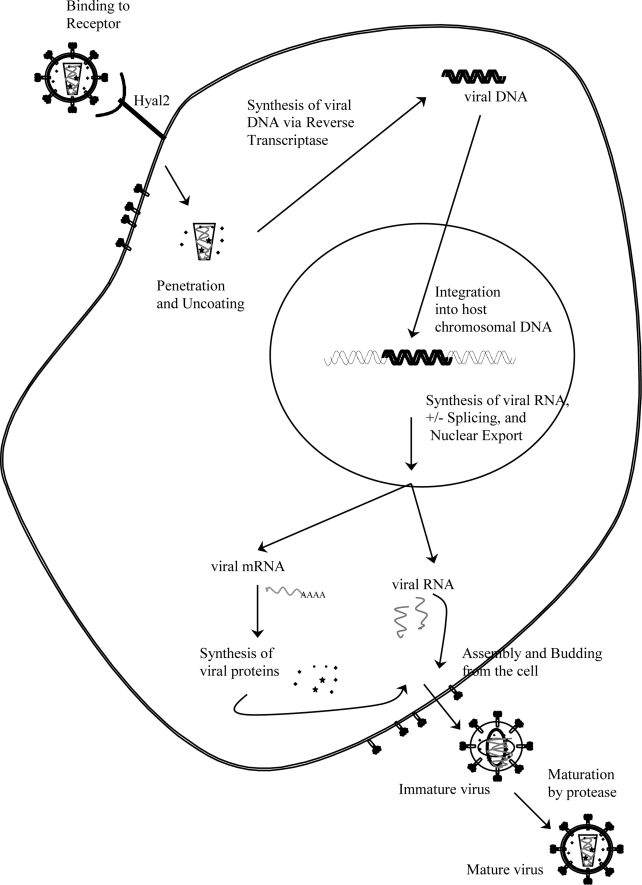
The retroviral life cycle. See text for details.

The lifecycle of JSRV presumably resembles that of other retroviruses (Figure 3). First, the virion makes contact with the host cell via the envelope protein that binds to its receptor on the target cell. The initial contact event involves the SU glycoprotein of Env interacting with its cellular receptor, followed by membrane fusion (at the plasma membrane or in lysozomes, depending on the virus) likely mediated by the TM subunit [17]. For JSRV, entry is restricted to the cells that express a functional receptor for the virus. It has been demonstrated that the membrane fusion and cell entry mediated by JSRV Env protein is pH-dependent [18,19]. The JSRV receptor has been identified as the glycosylphosphatidylinositol-anchored protein hyaluronidase-2 (Hyal2) [20]. After virus Env/receptor recognition and the subsequent penetration of the virion, the single stranded viral RNA genome is reverse transcribed by the viral enzyme reverse transcriptase to yield a double stranded DNA version of the viral genome. During this process, the U3 and U5 regions are duplicated to produce two complete long terminal repeats (LTRs) in the same orientation at each end of the viral DNA (Figure 1). As for all retroviruses, each LTR contains the promoter and enhancer regions necessary to drive transcription, as well as the polyadenylation signal for “termination” of the viral transcript [21,22]. The enhancer sequences in the LTR are a second major determinant of retroviral tissue tropism in addition to the viral envelope [23,24]. After reverse transcription for many viruses, the viral DNA enters the nucleus after mitosis. Once in the nucleus, viral integrase inserts the viral DNA at multiple sites into the host genome, resulting in the integrated provirus. Proviral integration occurs at multiple sites in the host DNA. Reverse transcription and integration of this viral DNA into the genome are hallmark features of the *Retroviridae* family. Viral RNA transcription is carried out by host RNA polymerase II with initiation in the 5’ LTR and termination in the 3’ LTR, resulting in full-length viral RNA. Some viral transcripts are spliced into subgenomic viral mRNA and translated into proteins (e.g., Env) by cellular protein synthesis machinery; other full-length transcripts are incorporated into virions or they are translated into other viral proteins (e.g., Gag and Pol). In general, the export of incompletely or unspliced RNAs from the nucleus is restricted by the cell; therefore nuclear export of unspliced retroviral RNAs requires specific mechanisms (discussed in further detail below). Retroviral virions are assembled into immature particles before or during budding from the host cell, although the site of viral core assembly is different for different viruses (intracellular *versus* at the plasma membrane). For JSRV and other betaretroviruses, initial assembly of the core occurs in an intracellular cytoplasmic (perinuclear) compartment [13], followed by transport of the cores to the plasma membrane for budding. Once the immature virion is released from the cell, maturation of the particle is accomplished by viral protease (PR) cleavage of the unprocessed viral polyprotein precursors [17].

## 3. Historical Perspective—JSRV the Causative Agent of OPA

**Figure 4:**
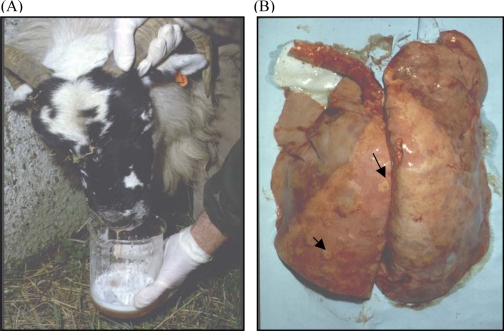
Clinical characteristics of ovine pulmonary adenocarcinoma. (**A**) OPA affected animals develop progressive respiratory distress, reflected by the accumulation of fluid within the respiratory tract. (**B**) Sheep lungs from an animal with OPA. The lungs display lesions (arrowed) characteristic of OPA as well as exudation of foamy lung fluid from the trachea. Adapted from [36].

Initial experiments on OPA lung fluid indicated that the viral agent of this disease was a retrovirus. Reverse transcriptase activity could be detected in OPA lung fluid [25–27]. Further evidence for a retroviral association with OPA lung fluid, was the observation that antibodies for two other retroviruses, mouse mammary tumor virus (MMTV) and Mason-Pfizer monkey virus (MPMV) cross-reacted with OPA tumors [28]. A landmark discovery was the generation of cDNA clones made from reverse transcribed RNA from partially purified virus from lung fluid. This allowed the deduction of a complete nucleotide sequence for a novel virus, jaagsiekte sheep retrovirus (JSRV) [29,30]. Jaagsiekte is the Africans name for OPA (Figure 4). The sequence of JSRV was typical of betaretroviruses (Figure 1), and it was phylogenetically related to the betaretroviruses MMTV and MPMV, consistent with the antibody cross-reactivity. However, attempts to demonstrate infectivity or oncogenicity from a full-length cDNA were unsuccessful, most likely because a tissue culture system to propagate the virus was not yet available. Nevertheless, the availability of the sequence allowed generation of important hybridization and immunological probes [10,31,32].

## 4. Endogenous and Exogenous JSRV

One important finding was the identification of multiple copies of highly related endogenous JSRV-like proviruses in the sheep genome [30,33–35]. This was not too surprising since the genomes of virtually all vertebrates, especially mammals, have been colonized by retroviral infection during evolution. These integrated retroviral DNAs are referred to as endogenous retroviruses [34,37]. In contrast to exogenous retroviruses, endogenous retroviral sequences (ERVs) are typically replication defective due to point mutations or deletions in their coding and/or regulatory regions; this may reflect selection during evolution against the deleterious effects of replication-competent retroviruses in their hosts [37]. The identification of endogenous JSRV (enJSRV) sequences was important since it meant molecular studies on JSRV infection must differentiate between endogenous and exogenous JSRV (exJSRV) sequences. Distinguishing enJSRV and exJSRV RNA or DNA sequences was possible because these two counterparts have different hybridization and restriction endonuclease restriction patterns [10]. Thus, it was possible to show that all OPA tumors contained exJSRV DNA, and the tumor cells stained positively with polyclonal antibodies raised against JSRV Gag protein [38]. Interestingly, the only cells in an OPA-affected animal in which JSRV protein can be reliably detected are the OPA tumors themselves [38]. Though enJSRV *env* sequences are not oncogenic, it should be noted that the expression of enJSRV proteins have been shown to block infection by exogenous JSRV [39–42]. The mechanisms by which enJSRVs interfere within exogenous JSRV infection are discussed below.

## 5. Isolation of an Infectious and Oncogenic Molecular Clone of JSRV

**Figure 5:**
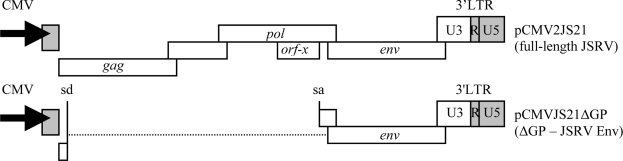
Molecular clone of the exogenous JSRV21 isolate. pJSRV21 is a plasmid containing the full-length integrated proviral sequences of JSRV21. The JSRV 5’ LTR was replaced with the CMV immediate early promoter (pCMV2JS21) in order to drive robust expression of the JSRV21 sequences in a multitude of cell lines that normally do not display high transcription of the JSRV LTR [21]. pCMVJS21ΔGP is a derivative construct of pCMV2JS21 in which the reading frames for Gag/Pol have been removed and expresses only the JSRV Env. The *env* gene splice donor (sd) and splice acceptor (sa) sites are retained in plasmid ΔGP, necessary for efficient Env expression. Adapted from [43].

The deduction of the JSRV sequence was a major advance that opened the way to molecular studies of JSRV/OPA. Importantly, it led to the identification of exogenous JSRV-specific restriction endonuclease polymorphisms [10,31] and the development of PCR-based diagnostic tools for exogenous JSRV-specific DNA [32]. These advances aided attempts to obtain an infectious molecular clone of JSRV. We used a sib-selection strategy to clone a full-length JSRV provirus from tumor DNA from a natural OPA case from the U.K. [2]. A lambda phage library was constructed from this tumor DNA and screened by both PCR analysis and by plaque hybridizations using JSRV DNA probes. Plaque DNAs were digested by a restriction endonuclease that distinguishes exJSRV from enJSRV and a lambda phage with a complete integrated JSRV provirus was identified (λJSRV21). The insert from λJSRV21 was then subcloned into a plasmid to give pJSRV21 (Figure 5). A technique was then developed to facilitate production of infectious JSRV particles from the molecular clone. This was accomplished by replacing the JSRV promoter/enhancers in the upstream LTR of pJSRV21 with the human cytomegalovirus (CMV) immediate early promoter, to give pCMV2JS21 (Figure 5). Virus particles were efficiently produced from pCMV2JS21 when transiently transfected in human 293T cells [2]. Importantly, when virus particles from pCMV2JS21 were concentrated and inoculated intratracheally into newborn lambs, 50% of test subjects (two out of four) developed OPA at four months [2]. This demonstrated that JSRV21 is an infectious and oncogenic molecular clone of JSRV and that this virus is necessary and sufficient to induce OPA. Another infectious and oncogenic molecular clone of JSRV has also been isolated [1].

## 6. Retroviral Oncogenesis

In considering JSRV induced-transformation, the known mechanisms of retroviral oncogenesis will first be discussed. Oncogenic retroviruses can be classified according to their mechanisms and rapidity of tumorgenesis: acute transforming and non-acute transforming retroviruses.

### 6.1. Acute Transforming Retroviruses

Acute transforming retroviruses induce neoplasms rapidly and have been identified in a wide range of animals such as birds, cats, mice, and primates [3]. The tumors induced by these viruses are generally polyclonal. Additionally, many of these viruses can morphologically transform cells in culture [3]. This high tumorigenicity results from the fact that these viruses carry a viral oncogene [24]. Viral oncogenes typically originated from normal cell genes that were incorporated into the retroviral genomes by recombination [44]. The cellular genes, called proto-oncogenes, are widely conserved in evolution and usually function in positive stimulation of normal cell growth and differentiation. During the capture process, the coding sequences for the viral oncogene typically are altered in comparison to the cellular proto-oncogene through base substitutions, domain deletions, and/or fusion to viral sequences [45]. As a result, viral oncogene proteins can induce uncontrollable cell growth. However, during the viral capture of a cellular proto-oncogene into its genome, the virus must accommodate this acquisition at the expense of one or more viral genes since there is an upper limit to the length of viral RNA that can be carried by a retroviral particle (*ca.* 8–10 Kb, [14]). As a result, almost all acute transforming retroviruses are replication deficient, and depend on co-infection with a related replication-competent retrovirus to provide viral proteins for production of infectious virus particles [3].

There are more than 25 distinct retroviral oncogenes carried by different acute transforming retroviruses, all of which are potent inducers of neoplastic transformation. Many oncogenic proteins function in signal transduction pathways involved in cellular responses to extracellular stimuli. Many oncogenes are protein kinases, while others are cell surface growth factor receptors and targets of second messengers. For example, the protein encoded by the *erb*B oncogene of the acute transforming avian erythroblastosis-ES4 virus is derived from the epidermal growth factor receptor (EGFR) [46]. EGFR is a member of the receptor protein tyrosine kinase family. The *erb*B oncogene product is a truncated version of the EGF receptor that retains the tyrosine kinase and transmembrane domains but lacks the amino terminal extracellular ligand binding domain and a C-terminal negative regulatory domain [46]. As a result *v*-*erb*B signals for growth in a ligand-independent unregulated manner.

Key cellular proteins involved in signal transduction and cell growth that were originally discovered as proto-oncogene counterparts of retroviral oncogenes are numerous. This includes the *ras* [47] and *raf* [44] proto-oncogenes, both are frequently mutated in many human cancers [47]. Two other onco-proteins, Akt and phosphatidylinositol 3-kinase (PI3K), are also frequently mutated in cancers; both were first identified as viral oncogenes. The cellular Akt protein is the analogue of the viral *v-Akt* oncogene of AKT8 virus that causes murine T cell lymphoma [48], and PI3K has also been captured as a retroviral oncogene in the acutely transforming retrovirus avian sarcoma virus 16 (ASV16)—*v-p3K* oncogene [49,50]. The *v-myc* oncogene (originally discovered in avian MC29 virus) was derived from the *c-myc* proto-oncogene [51]. Examples of oncogenes that encode nuclear proteins include *mye*, *myb*, *fos*, and *jun* [52–54].

### 6.2. Non-acute Retroviruses

Non-acute retroviruses are much less efficient and relatively slower inducers of tumors than the acutely transforming retroviruses [55]. They do not transform cells in culture [55], and they do not carry viral oncogenes. Rather, the mechanism for oncogenesis is insertional activation of proto-oncogenes [56]. Unlike most acute transforming retroviruses, these viruses are replication-competent [24]. During retroviral replication, integration of proviral DNA occurs at many sites (almost randomly) with respect to the host chromosomal DNA [57,58]. Thus, the chromosomal sites of proviral insertion will likely be different for each infected cell. However, in tumors induced by non-acute retroviruses, proviral insertions often can be detected in the vicinity of the same cellular proto-oncogene in different tumors. The strong enhancers and/or promoters in the proviral LTR promote the over-expression of the adjacent proto-oncogene [3], leading to enhanced cell growth. The over-expression of a proto-oncogene due to the proviral integration typically occurs by one of two mechanisms. In the first, called promoter insertion, transcription initiates in the viral downstream LTR, followed by read-through into the downstream proto-oncogene sequence [59]. The over-expression of the adjacent proto-oncogene may also result from initiation in the upstream LTR, followed by readthrough into the proto-oncogene. In the second mechanism, called enhancer activation, transcriptional enhancers in the viral LTR activate over-expression from the endogenous promoter of the neighboring proto-oncogene [60,61]. Insertional activation of proto-oncogenes at least partially accounts for the longer time course of tumor induction by non-acute retroviruses. Multiple rounds of infection, (and additional time) are required in an infected animal until insertion next to a proto-oncogene occurs in one cell [3]. It is that cell that will develop into the tumor. The discovery of the insertional activation mechanism was made in B-lymphomas induced by Avian leukosis virus (ALV), a replication-competent non-acute retrovirus. The B-lymphomas were found to contain ALV proviruses inserted upstream of the c-*myc* proto-oncogene with readthrough from the provirus into the c-*myc* gene (promoter insertion) [51].

## 7. JSRV Tumorigenesis

The isolation of a complete, infectious molecular clone of an exogenous JSRV provirus, and the ability of the resulting JSRV virions to induce lung cancer in sheep, demonstrated that JSRV is the causative agent of OPA [1,2]. With this established, the mechanism of JSRV-mediated oncogenesis has been of great interest. Initially, it was important to determine if JSRV is an acute transforming retrovirus, or a non-acute retrovirus. The biological and molecular characteristics of JSRV oncogenesis gave conflicting indications of whether JSRV is an acute transforming or non-acute retrovirus. When OPA lung fluid from an affected animal is concentrated and injected intratracheally into newborn lambs, the mean time to development of end-stage OPA is 3–6 weeks, with disease evident in as little as 10 days [3,11,62,63]. The rapid oncogenesis induced by JSRV would suggest that JSRV carries an oncogene (*i.e.*, is an acute transforming retrovirus). However inspection of the genome sequences did not reveal any obvious oncogenes or sequences with homology to known cellular genes, more characteristic of a non-acute retrovirus [3]. In sheep with OPA, the lungs typically contain multi-focal lesions, which are more typical of tumors induced by an acute transforming retrovirus [62]. Non-acute retroviruses tend to induce monoclonal or oligoclonal tumors due to the low probability of insertional activation of a cellular proto-oncogene [4,24,56].

### 7.1. JSRV Env is an Oncogene

**Figure 6:**
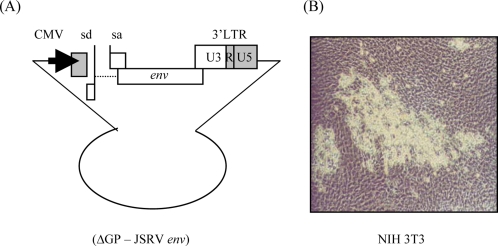
Transformation by the JSRV Env in NIH 3T3 fibroblasts. (**A**) The JSRV *env* expression plasmid (ΔGP) is driven by the constitutively active CMV promoter. (**B**) Demonstration of JSRV Env induced foci formation in NIH 3T3 cells transfected with ΔGP. Adapted from [43].

To address the possible mechanisms of JSRV oncogenesis, we tested if JSRV might carry an oncogene. This was done by investigating whether the JSRV genome can morphologically transform cells in culture. The initial transformation experiments were performed using DNA transfection of murine NIH 3T3 cells. Because NIH 3T3 cells are contact-inhibited, but sensitive to transformation by retroviral oncogenes and activated cellular proto-oncogenes, they have been commonly used to test for the presence of viral oncogenes or activated proto-oncogenes [64,65]. To test the JSRV genome for transforming potential, a CMV-driven JSRV21 expression plasmid was used (Figure 5), since the JSRV LTR has very low transcriptional activity in NIH 3T3 cells [21]. Transfection of JSRV21 DNA into NIH 3T3 fibroblasts resulted in the appearance of transformed foci, and the foci contained JSRV DNA [43]. This indicated that JSRV contains a gene with potential to transform NIH 3T3 cells and strongly suggested that it carries an oncogene. Subsequent experiments indicated that expression of the JSRV *env* gene alone (ΔGP, Figure 6) was necessary and sufficient for inducing transformation of NIH 3T3 cells [43]. The oncogenic capacity of the JSRV Env has also been demonstrated *in vivo*, as discussed in section 11.

The capacity of JSRV *env* to transform cells in culture and induce tumors *in vivo*, and the rapid rate of JSRV induced OPA indicates that JSRV can be considered an acute transforming retrovirus [43,62]. However, unlike most acute transforming retroviruses, JSRV does not carry a host cell-derived oncogene, but rather its Env protein has transformation potential. The only other examples of retroviral envelope proteins functioning as oncogenes are from enzootic nasal tumor virus (ENTV), avian hemangioma retrovirus (AHV), and the replication-defective Friend spleen focus-forming virus (SFFV) [66–69]. For AHV, the Env protein has also been reported to induce proliferation of cultured cells [67], although no follow-up reports since the initial publication have appeared. For Friend SFFV a deleted version of a recombinant Env protein (gp55) can induce proliferation of erythroid cells in culture and in animals. SFFV gp55 binds to and activates the erythropoietin receptor, leading to the constitutive activation of signal transduction pathways downstream of the receptor [69–71]. SFFV gp55 also binds to the short form of the Stk/Ron receptor, and this results in the activation of downstream signaling pathways [72,73].

**Figure 7:**
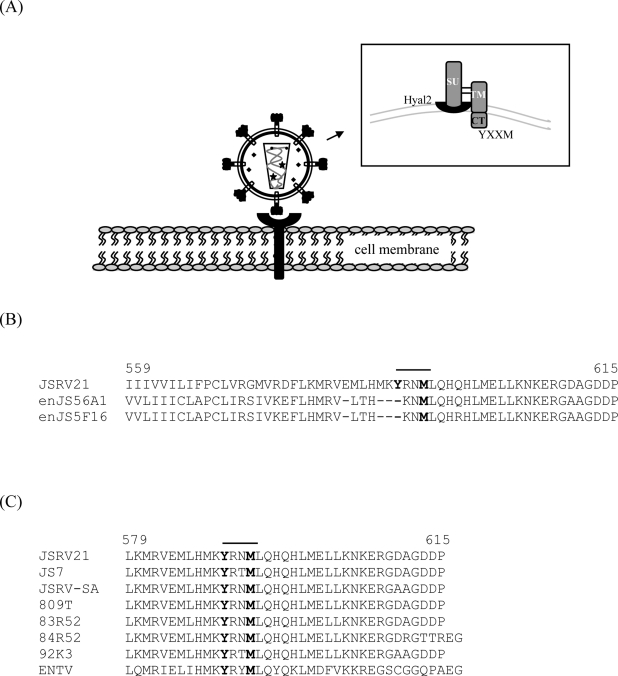
Schematic representation of the JSRV Env protein at the cell plasma membrane and sequence alignments of the Env cytoplasmic tail. (**A**) Hypothetical model of the JSRV Env (on a virion) with the JSRV receptor, Hyal2, and its likely positioning in the cellular membrane (insert). The JSRV Env is composed of two subunits, surface (SU) and transmembrane (TM). The cytoplasmic tail (CT) of the TM domain contains a YXXM motif, a putative PI3K/p85 binding site. (**B**) Alignment of the amino acid sequence of the TM region of the exogenous JSRV21 and the endogenous enJS56A1 and enJS5F16 clones. A dash (−) indicates lack of an amino acid. The amino acids of the putative PI3K docking site are in bold. (**C**) The putative PI3K docking site is conserved between various exJSRV isolates. Adapted from [75].

The JSRV Env is initially expressed as a membrane-spanning protein of 615 amino acids in length prior to maturation [1,2,30]. After cleavage by cellular furin protease, the Env protein is composed of N-terminal surface (SU) and C-terminal transmembrane (TM) domains, linked by disulfide bonds (Figure 7A). As with all retroviruses, the SU domain is at the surface of the virus and involved in receptor binding, while the TM anchors SU to the virus and is responsible for fusion of the viral and cellular membranes during infection. For JSRV, the TM domain of Env spans the lipid bilayer of the viral envelope, and has an internal tail region of 44 amino acids in length (Figure 7). It is very likely that, in infected or transformed cells, the JSRV SU protein is extracellular, anchored to the plasma membrane by TM, with the 44 amino acid tail in the cytoplasm—this 44 amino acid domain is referred to as the cytoplasmic tail. The cytoplasmic tail of Env contains the sequence YRNM; if the tyrosine (Y) is phosphorylated, the sequence could potentially function as a binding site for the PI3K/p85 regulatory subunit [74]. The molecular mechanism for transformation, involving the YXXM motif as well as the other Env protein domains, has been investigated extensively and is elaborated below.

### 7.2. JSRV Env Domains Involved in Transformation

*In vitro* transformation by the JSRV envelope has been studied in various cell lines, including NIH 3T3 murine fibroblasts [43,75], 208F rat fibroblasts [76,77], Rat6 fibroblast cells [43], DF-1 avian embryo fibroblasts [78,79], Madin-Darby canine kidney (MDCK) epithelial cells [80,81], rat kidney epithelial (RK3E) cells [82], and human bronchial BEAS-2B epithelial cells [83]. However, the details of JSRV Env transformation may differ between cell lines and culture conditions [75–79]. Deletion and/or mutation experiments have shown that the cytoplasmic tail of TM is essential for transformation [75,77,84,85]. The YXXM motif in the TM cytoplasmic tail is conserved between all transforming JSRV and ENTV strains, but it is absent in the Env proteins of non-transforming JSRV-related endogenous retroviruses of sheep (Figure 7B and C) [37]. This would be consistent with a model in which JSRV Env docks PI3K to the plasma membrane, leading to signaling through Akt and downstream pathways. Indeed, mutation of the tyrosine residue to phenylalanine or aspartic acid abolished Env transformation in NIH 3T3 cells [75,77], although in some other cell lines inhibition of transformation was not absolute [76,78,80,81]. Likewise, mutation of the methionine residue of the YXXM motif also abolished transformation in NIH 3T3 cells [75], although effects in other cell lines were variable [76–78]. Moreover, JSRV transformed cells generally show constitutive activation of Akt [79], and inhibitors of PI3K have been reported to inhibit JSRV transformation [82,85,86].

However, no tyrosine phosphorylation has been detected for the JSRV Env protein in transformed cells, although this could have been due to limits of sensitivity [76]. While the decrease in JSRV Env transformation by mutants in the YXXM motif, indicates that it is important for Env transformation, the results described above suggest the YXXM motif does not activate PI3K by binding to it. Pull-down type experiments also have not detected direct interaction between Env and PI3K [86,87]. Thus, the exact mechanism by which the YXXM motif participates in JSRV transformation remains to be determined [76,80]. The role of the PI3K/Akt signaling pathway in JSRV Env transformation is further described below.

While the cytoplasmic tail of TM is essential for JSRV transformation, it is possible that other domains of Env participate as well. Indeed, deletions or small insertions in the SU glycoprotein also abolished JSRV Env transformation in both NIH 3T3 and 208F fibroblasts [77]. Moreover, chimeras between the endogenous and exogenous JSRV Env, in which the TM cytoplasmic tail domain remained exogenous while the entire SU and extracellular/membrane-spanning region of TM was replaced with endogenous sequences, failed to transform NIH 3T3 cells [75]. Also, exchange of the amino acids in JSRV SU to those of endogenous JSRV-related Env amino acids also substantially reduced JSRV transformation in rodent fibroblasts [88]. We also found that the SU domain of Env functions independently of TM in transformation. Cotransfection of a transformation-defective Env mutant defective in SU with a TM mutant showed complementation for transformation in 208F cells. Thus efficient transformation by JSRV Env may require signals from both SU and TM [77].

Another mechanism for JSRV SU involvement in transformation has been described by Danilkovitch-Miagkova *et al.* [83], who studied BEAS-2B human bronchial epithelial cells. They showed that in these cells the receptor for JSRV, hyaluronidase 2 (Hyal2), binds the Stk/RON growth factor receptor. The Hyal2-RON complex has low Ron kinase activity; when JSRV Env is expressed, the Hyal2 is bound by the Env instead, freeing RON with a resulting increase in kinase activity. In these cells activation of RON activates both the PI3K/Akt and MAPK pathways [83].

SU also appears to influence JSRV transformation independent of Hyal2/RON as well. Rodent fibroblasts can be transformed by JSRV Env, but JSRV SU protein does not bind the mouse homologue of Hyal2. Furthermore, overexpression of mouse Hyal2 has no effect on JSRV Env transformation in NIH 3T3 or 208F cells [20,89,90]. In addition, Stk/RON is not expressed in fibroblasts [91]. One possibility for the involvement of the SU subunit in JSRV Env transformation is that SU transmits independent signals for transformation that collaborate with signals triggered by the TM domain, resulting in transformation.

### 7.3. Signaling Pathways in JSRV Env-Induced Transformation

**Figure 8:**
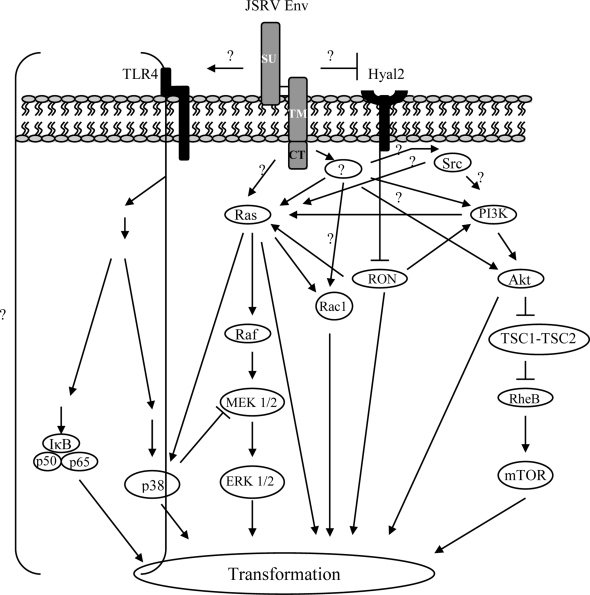
Signaling pathways involved in JSRV Env-induced cell transformation. Depending on the type of cell line studied, three main pathways have been shown to be activated in JSRV transformed cells; the PI3K/Akt pathway, the Hyal2-RON pathway, and the Ras-MEK-ERK pathway. Signaling through the TLR4-NFkB pathway is also depicted, since the JSRV Env interacts with TLR4, though activation of this pathway has yet to be formally shown. A fourth pathway; via signaling through Rac1, has recently been identified, but less is known about it [86]. Except for the Hyal2-RON pathway, how the JSRV Env engages the Ras-MEK-ERK or the PI3K/Akt pathways is not known. Possible interactions, or yet to be identified factors that may be involved in JSRV Env-mediated transformation, are indicated with question marks (?). Each pathway involved in JSRV Env-transformation leads to the activation of factors involved in cell survival, proliferation, apoptosis, and differentiation. See text for details.

Signal transduction pathways involved in JSRV transformation or tumorigenesis have been evaluated by studying the effects of specific inhibitors in transformation and by western blotting and immunohistochemistry for activated (phosphorylated) proteins in signaling cascades. Three signaling pathways have been reported to be involved in cell transformation by JSRV Env (Figure 8). The first is the PI3K-Akt pathway, well known as a determinant in cellular proliferation, survival, oncogenic transformation, and cancer development [92,93]. This pathway can be activated by several factors, such as growth factors, hormones, or cytokines, but also by mutations or overexpression of receptor or nonreceptor tyrosine kinases [92,93]. Activation (phosphorylation) of the downstream Akt kinase has been shown to be both dependent and independent of PI3K (Figure 8). Briefly, PI3K dependent activation of the Akt pathway is initiated by recruitment of PI3K to the cell membrane, e.g., by binding of the p85 subunit to a tyrosine-phosphorylated receptor tyrosine kinase via the SH2 domain. Membrane-bound PI3K generates phosphatidylinositol (3,4,5) tris phosphate (PIP3) from phosphatidylinositol (4,5) bis phosphate. PIP3, in turn, recruits phosphatidylinositol-dependent kinase 1 (PDK1) and Akt to the plasma membrane, whereupon PDK1 phosphorylates (activates) Akt. Activation of Akt independent of PI3K activation also occurs; one mechanism involves the secondary messenger cAMP, which triggers the phosphorylation of Akt by calcium/calmodulin dependent kinase (CaMKK) [94,95]. Activated Akt can phosphorylate several substrates involved in controlling cellular proliferation; one pathway leads to the activation of the mammalian target of rapamycin (mTOR), an important regulator of cell growth (Figure 8).

As described above, constitutive Akt activation has been observed in several cell lines transformed by JSRV [75,76,79,80], as well in OPA tumors [96]. Moreover, OPA-derived type II pneumocytes do not respond to EGF stimulation, an activator of the PI3K-Akt pathway, suggesting dysregulation of the Akt pathway by JSRV infection [96]. On the other hand, treatment of cells with the PI3K-inhibitors, LY294002 and wortmannin, drastically reduces Akt phosphorylation in JSRV Env transformed cells [43,76,79,80,97], indicating that PI3K-dependent Akt activation can occur in these cells. While the YXXM motif is important for transformation, it does not appear that this motif is directly binding and activating PI3K. The binding of JSRV TM and the p85 subunit of PI3K, or phosphorylation of the Y590 of the YXXM motif, has never been demonstrated, and mutations of the tyrosine residue of this motif did not completely abolish transformation in some cell lines [76,80]. Although a decrease in JSRV Env transformation was observed in these studies, mutations of the JSRV YXXM motif also did not abolish Akt phosphorylation [76,79,80]. Thus, JSRV Env might activate PI3K/Akt indirectly, possibly through as-yet unidentified cellular adaptor molecules or by other signaling pathways triggered by JSRV Env (Figure 8). NIH 3T3 cells deficient for the p85 subunit of PI3K can still be transformed by JSRV, indicating that PI3K is not essential for JSRV transformation [97]; however, even in this situation, Akt is still constitutively activated. Nevertheless, regardless of the dependence on PI3K, Akt activation is currently a hallmark of JSRV Env-mediated transformation [79,84]. Moreover, the downstream Akt substrate mTOR, has been shown to be involved in JSRV Env transformation of NIH 3T3 cells, as the mTOR inhibitor, rapamycin, partially inhibits Env transformation efficiency [82].

The second signaling pathway identified by which JSRV Env mediates cell transformation is the mitogen-activated protein kinase (MAPK) signaling pathways (Figure 8) [98]. A major pathway for activation begins with the Ras proteins that initiate a signaling cascade that eventually leads to translocation of activated MAPKs into the nucleus, where transcription factors involved with cellular division and proliferation are phosphorylated and activated [98]. There are three MAPK types: extracellular signal-regulated kinase 1 and 2 (ERK1/2), stress-activated protein kinase/c-Jun amino terminal kinase (SAPK/JNK), and MAPK p38 (Figure 8). Activation of the Ras protein (see above) stimulates phosphorylation of the MAPKK kinase (MAPKKK) Raf (Figure 8). Activated Raf protein in turn phosphorylates (activates) MAPK kinase (MAPKK) such as MEK1/2, which then phosphorylates MAPKs, such as ERK1/2. The Ras-Raf-MEK-ERK1/2 pathway has been shown to play the most critical roles in cell transformation in tumors by other oncogenic viruses, as well as in non viral cancers [99].

The involvement of the MAPK pathway in JSRV Env transformation was indicated by experiments using specific inhibitors of this pathway. The MEK1/2 inhibitor, PD98059, as well as an H/N-Ras inhibitor, FTI-277, strongly inhibited Env transformation of NIH 3T3 in a dose-dependent manner [82,85]. In RK3E rat kidney epithelial cells, PD98059 also strongly inhibited transformation, but FTI-277 only partially inhibited transformation. Thus, in the latter cells, signaling to MEK1/2 also occurred through other molecules than H/N Ras (e.g., K-Ras). In agreement with these studies, experimentally and naturally induced OPA tumors stained positive in immunohistochemistry for phosphorylated ERK1/2 [82,100]. Moreover, the MAPK component p38, that down-modulates signaling from MEK1/2 to ERK1/2 [101], negatively regulates JSRV transformation since treatment with the p38 inhibitor SB203580 increased JSRV-Env induced transformation [82]. Exactly how the JSRV Env activates the p38 pathway remains to be determined (Figure 8). Taken together, these results indicate the Ras-MEK-MAPK pathway is important in JSRV Env-mediated transformation.

A third potential mechanism implicated in JSRV Env-mediated transformation involves signaling through the JSRV receptor, Hayl2 (Figure 8). This mechanism was implicated by studies on the minimally transformed human bronchial cell line BEAS-2B, as described above [80,83,102]. Of particular importance, experiments using a dominant-negative kinase-dead RON mutant were able to block transformation by Env, indicating that the RON/Hyal2 pathway is critically important for JSRV Env transformation of BEAS-2B cells [83]. However, the Hyal2-RON interaction cannot account for JSRV transformation in all cells. This mechanism requires the binding of Env and Hyal2, which does not occur in rodent cells, since JSRV Env does not bind mouse Hyal2 nor does it facilitate entry of JSRV into murine cells [89]. Furthermore, the mouse ortholog of RON, stk, is not expressed in NIH 3T3 cells [91]. Nonetheless, this mechanism may be important for JSRV Env-mediated transformation of sheep type II pneumocytes, since they have functional JSRV receptors and express RON [4].

In addition to the three pathways described above, additional signaling pathways may be involved in JSRV transformation. Rassa *et al.* [103] have shown that the Env protein of MMTV binds the cellular Toll-like receptor 4 (TLR4) protein; TLR4 is a membrane-bound pathogen-associated molecular pattern (PAMP) receptor involved in innate immunity. Engagement of TLR4 by extracellular ligand (bacterial lipopolysaccharide) leads to a signaling cascade that activates the NFkappaB transcription factor as well as other signaling molecules (Figure 8). Binding of TLR4 by MMTV Env results in positive signaling in dendritic cells and B-lymphocytes [103,104], and it has been shown that this engagement is important for initial establishment of MMTV infection *in vivo*. Other retroviral envelope proteins have been found to bind TLR4, including those of Moloney murine leukemia virus [103] and JSRV [105]. Thus it is possible that signaling downstream of TLR4 by JSRV Env binding also contributes to JSRV transformation (Figure 8). Another signaling pathway that may be important for JSRV transformation involves the c-Src protein, a non-receptor tyrosine kinase [46]. Mutational analysis of the JSRV Env cytoplasmic tail suggested involvement of signaling through c-Src, and this was supported by treatment with the src inhibitor PP2 [85]. A possible role of c-Src in JSRV transformation has also been reported by Varela *et al.* [106].

Most of the *in vitro* experiments on JSRV transformation have been conducted on fibroblasts or epithelial cell lines grown in monolayer. However, the *in vivo* targets of JSRV oncogenesis are polarized epithelial cells (type II pneumocytes). We have employed the MDCK canine kidney epithelial cell line that forms hollow spheres of polarized cells when grown in 3D culture by embedding cells in an extracellular matrix preparation (matrigel) [81]. Expression of JSRV Env in MDCK cells grown in matrigel, results in aberrant sphere formation as well as enhanced cell replication rate. Interestingly, the signaling pathways involved in the transformation in 3D differ from those in monolayer. In particular, signaling through Akt and PI3K were important, while the Ras-MEK1/2-ERK1/2 pathway was not. Thus, it will be important to consider the differentiation state of the target cells when considering JSRV transformation.

Although the pathways discussed above have been identified as being activated in JSRV Env transformed cells, it is not known how JSRV Env activates the cell-signaling network (Figure 8). As one approach, we have employed a yeast two-hybrid assay to identify cellular proteins that bind with the CT domain of Env [107]. Two candidate cellular proteins have emerged so far, the regulatory subunit of ribonucleotide reductase (RRM2), and a little studied zinc finger protein Zfp111/rKr2 that contains transcriptional repressor domains [108].

## 8. Nuclear Export of Unspliced Retroviral RNA

### 8.1. Regulatory Proteins

As mentioned previously, full-length viral genomic RNAs must be exported from the nucleus for both packaging into newly synthesized virions and translation of Gag poly protein (Figure 3). However, cells have developed mechanisms to prevent the export of incompletely spliced or intron-containing cellular RNAs, because translation of these RNAs would likely result in dysfunctional proteins with possible detrimental cellular effects [109]. In order to achieve export of unspliced viral RNAs from the nucleus, retroviruses typically use one of two strategies [110]. For complex retroviruses, export of full-length RNAs is facilitated by a virally encoded *trans*-acting factor, which binds to a specific sequence or region of the RNA. The Rev protein encoded from HIV-1 typifies such nuclear mRNA-export factors. Rev is translated from a doubly spliced HIV-1 mRNA and its mRNA is exported from the nucleus by the same mechanism used by spliced cellular mRNAs [110]. Rev protein is then transported into the nucleus via a nuclear localization signal (NLS) [111], interacting with the nuclear import receptor, importin-β [112]. In the nucleus, Rev mediates the export of full-length HIV-1 RNAs by binding to the *cis*-acting Rev-responsive element (RRE) on the RNA located in the env gene [113–116]; Rev then interacts with the cellular nucleocytoplasmic-transport factor, Crm1 [117]. Crm1 facilitates nuclear export of proteins that contain a leucine-rich nuclear export signal (NES), also found in the HIV-1 Rev protein [118], thereby enabling export of Rev-bound HIV-1 RNAs [117,119]. An analogous mechanism is used by other retroviruses, including human T-cell leukemia virus (HTLV-1 and Rex [120]) and the betaretroviruses human endogenous retrovirus type K (HERV-K and Rec [121]) and mouse mammary tumor virus (MMTV and Rem [122,123]).

### 8.2. cis-Acting RNA Elements

Simple retroviruses encode only Gag, Pol and Env [14], but they must also export full-length viral RNAs. Since they do not encode Rev-like proteins, they recruit host cellular factors to mediate export. The best-known example is a *cis*-acting constitutive transport element (CTE) found at the 3’ end of Mason-Pfizer monkey virus (MPMV) RNA (also a betaretrovirus). The CTE of MPMV is a highly ordered RNA stem-loop structure [124] that specifically binds the cellular Tap (NXF1) protein [125], the major nuclear export receptor for cellular mRNAs [110]. The binding of Tap to the CTE of MPMV, in conjunction with the Tap cofactor, p15 (NXT1) [126] facilitates export of unspliced MPMV RNAs from the nucleus. Another *cis*-acting element with export activity has been identified; the direct repeat regions (DRs) of Rous sarcoma virus (RSV) and Rous-associated virus type-2 (RAV-2) [127,128].

### 8.3. JSRV Rej and Expression of Unspliced Viral RNA

**Figure 9:**
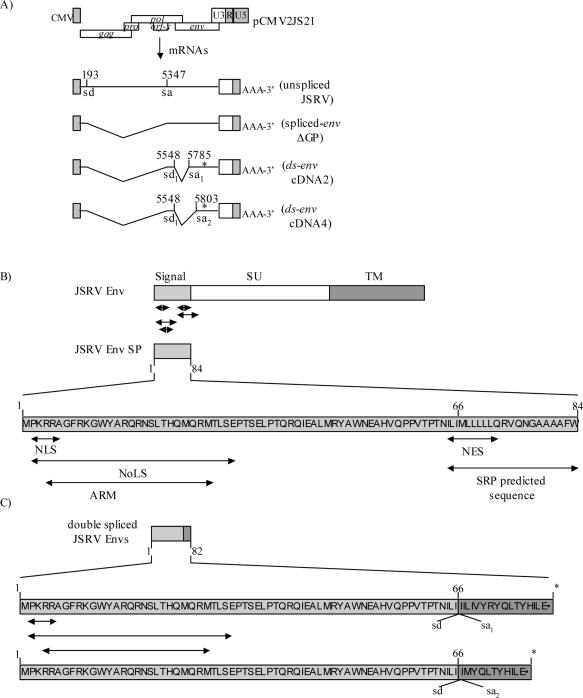
Schematic representation of the alternatively spliced transcripts within the JSRV *env* gene and the domains located within the Env signal peptide region. (**A**) Structure of doubly spliced *env* mRNAs with the *env* splice donor (sd) and splice acceptor (sa) nucleotides indicated. The locations of stop codons are indicated by asterisks. (**B**) Domain structures of the signal peptide of the JSRV Env. The JSRV Env signal peptide sequence is diagramed, and the locations of predicted sequence motifs are indicated by arrows under the diagram. (**C**) The putative translation products of the doubly spliced JSRV *env* transcripts. The locations of the alternative splice donor and splice acceptor sites are indicated, as well as the location of the stop codon (asterisks) within the second exons (shaded) [129].

We—and others—have recently shown that JSRV encodes an additional factor, named Rej or JSRV Env signal peptide (SP) required for synthesis of Gag polyprotein [129,130]. Rej is analogous to MMTV Rem, and may arise from one of two sources—a doubly spliced mRNA or from singly spliced Env mRNA (Figure 9). Functional Rej protein may consist of the cleaved signal peptide from Env polyprotein [129,130], or the translation product of the doubly spliced mRNA [129]. Rej protein contains motifs that are characteristic of the small rev-like proteins including putative nuclear and nucleolar localization signals, an arginine-rich motif and nuclear export signals (Figure 9). The translation product of the doubly spliced mRNA would lack C-terminal motifs (e.g., the nuclear export signal), but still contain the other motifs, and we have found that Rej translated from doubly spliced mRNA is functional [129]. In 293T cells, Rej shows modest enhancement in exporting unspliced viral RNA to the cytoplasm, but in other cells it does not. Rather, it is essential for efficient translation of unspliced viral RNA in all cell lines [129]. Thus, a primary mechanism of Rej action is enhancement of translation of unspliced viral RNA. The exact mechanism by which Rej functions to facilitate translation remains to be determined. The fact that Rej is not required for cytoplasmic export of unspliced viral RNA in most cell lines also indicates that JSRV RNA must carry a CTE. Indeed, we have found a composite cis-acting RNA element in the 3’ end of the JSRV *env* gene that contains 1) a CTE that facilitates export of full-length viral RNAs in most cells, and 2) a Rej-binding element (RejRE) that facilitates export of unspliced viral RNA in 293T cells and that is required for translation of unspliced viral mRNA in all cells [131]. This composite element has been termed the JSRV regulator of export and expression (JREE) [131]. Thus JSRV employs a unique strategy for viral RNA export and translation, using both a CTE and an accessory protein Rej.

## 9. Restriction of JSRV by Endogenous JSRV Sequences

JSRV replication *in vivo* is influenced by the interplay of enJSRV proteins and exogenous JSRV that can result in interference of JSRV infection. One example is receptor interference [42] in which expression of endogenous JSRV Env binds Hyal2 in order to block infection by exogenous JSRV. Another way enJSRVs interfere with the exogenous JSRV is through endogenous Gag proteins blocking assembly and release of exogenous JSRV [39,41]. The ability of the expressed endogenous JSRV proviral proteins to interfere with the JSRV lifecycle have become an interesting research area, due to the potential for disrupting the pathological function of JSRV in tissues where enJSRV are abundantly expressed (female reproductive tract). This may have driven the tropism of exogenous JSRV to tissues where enJSRVs are not expressed (e.g., the respiratory tract) [132].

## 10. Transcriptional Specificity of JSRV

JSRV is unique among retroviruses because it transforms the alveolar type II cells and the nonciliated bronchiolar cells (Clara cells) of the lungs. In studies investigating the cell specificity of JSRV expression, Palmarini *et al.* showed that the JSRV LTR is preferentially active in cell lines derived from type II pneumocytes and Clara cells (MLE-15 and mtCC1-2 respectively) [21]. The U3 sequences of the JSRV LTR has binding motifs for hepatocyte nuclear factor 3 (HNF-3/Fox-a2), a transcription factor involved in lung-specific gene expression, and exogenous HNF-3 is able to enhance the expression of the JSRV LTR in NIH 3T3 cells that normally show minimal enhancer activity for the LTR [21].

The JSRV LTR has two HNF-3 binding sites, and electrophoretic mobility shift assays (EMSAs) indicated that nuclear extracts from MLE-15, but not NIH 3T3, cells formed complexes with an oligonucleotide encompassing these sites [133]. Thus the JSRV LTR is activated by the lung-specific transcription factor HNF-3β in MLE-15 cells, suggesting that the JSRV tropism to the lungs is determined by the transcriptional specificity of the LTR [133].

The tissue specificity of the JSRV LTR was further demonstrated when JSRV U3 enhancer sequences were inserted into a Moloney murine leukemia virus (M-MuLV) LTR lacking its own enhancers [134]. M-MuLV driven by this chimeric LTR showed enhanced infectivity in lung epithelial cells that otherwise were not permissive for M-MuLV infection [134]. Two additional motifs in the JSRV LTR that bind C/EBP and NF-1 isoforms were later shown to be important for LTR activity as well [135].

Comparison of the LTR enhancer sequences of JSRV to those of the closely related ENTV and endogenous sheep retrovirus (enJSRV) LTRs have provided insights into the basis for tissue-specific expression [136]. JSRV and ENTV induce lung and nasal adenocarcinomas respectively, with enJSRVs primarily expressed in the female reproductive tract [42]. All three independent JSRV isolates have LTRs that contain the two predicted HNF-3 binding sites, while the ENTV and enJSRV LTRs do not [136]. In addition, the C/EBP binding site is interrupted in the enJSRV LTRs, but conserved in the ENTV LTR [136].

The *in vivo* tissue specificity of the JSRV LTR has also been investigated [137,138]. Dakessian *et al.* [137] generated transgenic mice carrying the bacterial beta-galactosidase gene driven by the JSRV LTR [137]. F1 animals from two transgenic lines showed transgene expression only in lung type II pneumocytes, but somewhat surprisingly not in Clara cells. Likewise, expression was not detected in bronchiolo-alveolar stem cells (BASC) that have been proposed as target cells for lung adenocarcinomagenesis. Thus the JSRV LTR is specifically active in type II pneumocytes in the mouse lung, which is consistent with the fact that JSRV-induced OPA tumors in sheep largely have phenotypic markers of type II pneumocytes. While the specificity for the JSRV LTR is preferential for the lungs, vectors driven by the JSRV LTR are also expressed in other tissues [138].

## 11. Small Animal Models for OPA

To further understand JSRV oncogenesis, several approaches have been made to obtain small animal models of OPA. A JSRV vector that only expressed *env* also induced lung cancer in sheep [139], and mice carrying JSRV transgenes also develop tumors—lipomas or lung adenocarcinomas [140,141]. For example, Dakessian *et al.* [141] generated a transgene expressing an epitope-tagged JSRV Env protein under control of the lung-specific surfactant A (SPA) promoter. In these studies, transgenic mice containing the SPA-Env-HA transgene showed low efficiency but specific expression in the lung, and the transgene also induced tumors (lipomas) in some animals [141]. More recently the lung-specific surfactant protein C (SPC) promoter was also used to drive expression of JSRV Env in transgenic mice, and these animals developed spontaneous lung tumors [140]. The tumors in these mice resembled OPA.

JSRV-induced disease has also been recapitulated in mice using viral vectors. Wootton *et al.* [142] used a replication defective adeno-associated virus (AAV) vector to express JSRV Env in the lungs of mice. In these experiments, immunodeficient mice infected intranasally with AAV6 vectors expressing Env developed lung tumors in 2–6 months [142]. The tumors closely resembled JSRV-induced OPA. These experiments indicated that JSRV Env dictates the specificity of the tumors, since only lung tumors developed, even though the AAV vector transduced a large variety of cells and tissues in the airways of the infected mice [142]. The Env protein of enzootic nasal tumor virus (ENTV), a close relative of JSRV that encodes a similar transforming Env protein which induces enzootic nasal adenocarcinoma (ENA), shows the same properties *in vitro* [66,68,86] and *in vivo* [142]. On the other hand, the same AAV vectors expressing the ENTV Env protein also induced bronchiolo-alveolar tumors in the lungs, and not tumors of the nasal epithelium, even though ENTV induces adenocarcinomas of the nasal epithelium. Thus, other factors in addition to Env mediate the tissue specificity of ENTV. Examination of the signaling pathways in ENTV and JSRV-transformed cells indicated that they are quite similar [8,86,143]. It seems possible that differential transcriptional specificity of the ENTV *versus* JSRV LTRs could be at least one of these factors.

## 12. Conclusions

Jaagsiekte sheep retrovirus has provided several unique insights into retroviruses and oncogenesis. No other viruses have been associated with lung cancer, one of the most common causes of cancer mortality in humans. Thus study of JSRV may provide insights into lung carcinogenesis. JSRV is also unique among retroviruses in that it is an acute transforming virus whose oncogene is its Envelope structural protein. Structure-function studies have identified activated signaling pathways involved in oncogenic transformation, most notably the PI3K-Akt-mTOR and Ras-Raf-MEK-MAPK pathways. As for many oncogenes, multiple pathways and mechanisms are likely to be activated. However, the mechanism(s) by which Env activates these pathways remains to be determined. Identification of cellular proteins that interact with the JSRV cytoplasmic tail will be key to understanding the mechanisms. Ultimately, it will be important to characterize JSRV transformation in differentiated lung epithelial cells, although maintenance of primary lung epithelial cells in their differentiated state is challenging. Studies of JSRV transformation in epithelial cell lines that can polarize, have been informative, and future experiments on primary cells or cell lines derived from lung epithelial cells will be key. Some of the signaling pathways and cellular proteins involved in JSRV oncogenesis may also be important for development of human lung adenocarcinoma.

Another unique feature of JSRV biology is that the virus shows transcriptional specificity for lung epithelial cells. This is unusual among retroviruses that typically have enhancers that promote transcription in multiple cell types. In contrast, in animals with OPA, the great majority of cells that express the virus are the tumor cells, while expression in other normal cells is rarely observed. It is interesting to compare the transcriptional specificity and growth-promoting potential of the Env protein of JSRV to enJSR*Vs.* The enJSRVs provide a view of the progenitor virus to JSRV, since they integrated into the sheep germline many generations ago, after which mutations are infrequent. The enJSRVs are not specific to lung epithelial cells, and they do not have cell transformation potential. One possible interpretation is that the JSRV has relatively recently evolved from an enJSRV-like virus to occupy a new biological niche: Infection of lung epithelial cells, and spread from animal to animal by aerosols. This adaptation would require transcriptional specificity for lung epithelial cells. In addition, since these cells are not normally cycling, and efficient retroviral infection and production generally require cells to be actively dividing, acquisition of the ability of JSRV Env to induce cell division may also be essential for viral replication in lung epithelial cells. Thus the oncogenic potential of JSRV may be a by-product of a requirement to induce non-cycling lung epithelial cells to divide.
